# Development and validation of instrument for ergonomic evaluation of tablet arm chairs

**DOI:** 10.17179/excli2016-568

**Published:** 2016-11-07

**Authors:** Adriana Seára Tirloni, Diogo Cunha dos Reis, Antonio Cezar Bornia, Dalton Francisco de Andrade, Adriano Ferreti Borgatto, Antônio Renato Pereira Moro

**Affiliations:** 1 Technological Center, Federal University at Santa Catarina, SC, Brazil; 2 Biomechanic’s Laboratory, CDS, Federal University at Santa Catarina, SC, Brazil

**Keywords:** school furniture, ergonomics, seated posture, item response theory

## Abstract

The purpose of this study was to develop and validate an evaluation instrument for tablet arm chairs based on ergonomic requirements, focused on user perceptions and using Item Response Theory (IRT). This exploratory study involved 1,633 participants (university students and professors) in four steps: a pilot study (n=26), semantic validation (n=430), content validation (n=11) and construct validation (n=1,166). Samejima's graded response model was applied to validate the instrument. The results showed that all the steps (theoretical and practical) of the instrument's development and validation processes were successful and that the group of remaining items (n=45) had a high consistency (0.95). This instrument can be used in the furniture industry by engineers and product designers and in the purchasing process of tablet arm chairs for schools, universities and auditoriums.

## Introduction

School is a very important part of a child's life, with children spending about six hours per day there, and 60-80 % of that time spent in a classroom (Savanur et al., 2007[42]). Marques et al. (2010[29]) highlight that maintaining a seated posture for more than four hours poses a risk to the musculoskeletal system. Considering this situation, and the possibility that inadequate school furniture is used, it is probable that postural alterations take place and problems develop in children's musculoskeletal systems, which not only affect students' health, but also their school performance (Castellucci et al., 2009[8]; Reis et al., 2012[39]).

Since schooling begins in early childhood and extends through to adulthood, companies must develop furniture that is suitable for different age groups. A variety of studies have indicated discordance between the students' anthropometric characteristics and the dimensions of school furniture (Chung and Wong, 2007[10]; Dianat et al., 2013[13]; Gouvali and Boudolos, 2006[17]; Jung, 2005[21]; Parcells et al., 1999[35]; Thariq et al., 2010[46]). This discrepancy leads to discomfort for students of different age groups and levels of schooling (Rego and Scartoni, 2008[38]; Murphy et al., 2004[32]). According to Khanam et al. (2006[23]), the comfort and functional utility of school furniture depend on its physical design in relation to the human body's physical structure and biomechanics.

**Figure 1:**
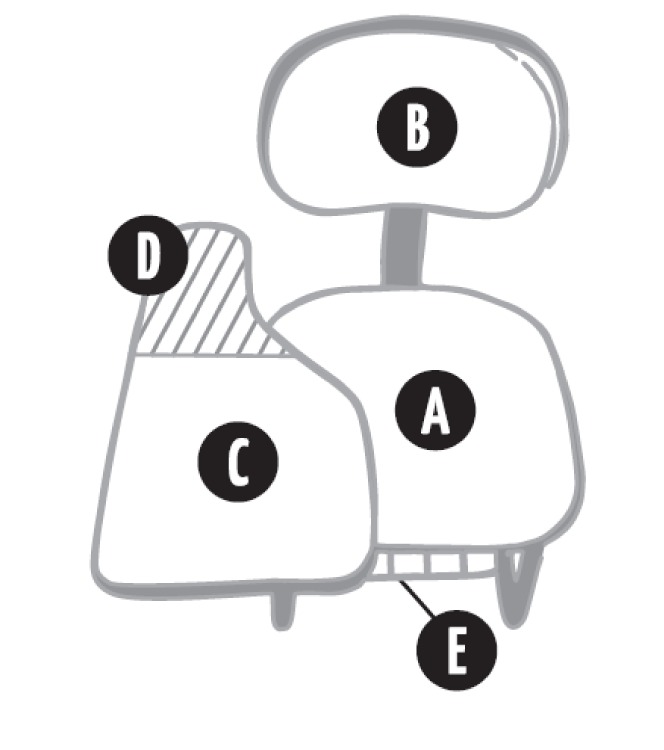
Tablet arm chair and its subsystems: (A) seat, (B) backrest, (C) tablet arm, (D) tablet arm extension, (E) material holder

Various international standards for school furniture establish ranges of body height of users and indicate the best respective sizes for a desk and chair, regardless of the school level: ISO 5970:1979 Furniture - Chairs and tables for educational institutions - Functional sizes (International Organization for Standardization, 1979[20]); EN 1729-1:2006 Furniture - Chair and tables for educational institutions - part 1: Functional dimensions (European Committee for Standardization, 2006[16]). In Brazil, there is a technical norm based on these international standards, ABNT 14006 - School furniture - Chairs and tables for educational institutions (Associação Brasileira de Normas Técnicas, 2008[4]), and these technical specifications are used to guide manufacturers and buyers of tablet arm chairs (Figure 1(Fig. 1)).

It is noteworthy that tablet arm chairs were made with chairs from the production lines of manufacturing companies, adapted with a tablet arm and a material holder (Ministério da Educação e Cultura, Brazil, 1982[31]). According to Soares (1998[43]), there are few criteria defined for the design of tablet arm chairs, and the measures furnished by the literature are insufficient for designing them. Tunay and Melemez (2008[48]) affirm that the protection of the physical and mental health of product users depends on the use of equipment that has been produced according to ergonomic principles. 

Therefore, it is important to build an instrument for measuring the suitability of ergonomic requirements for tablet arm chairs to their users. The suitability is related to ergonomic requirements and it is a latent trait, a variable that cannot be directly measured, so it was used Item Response Theory (IRT). According to Edelen and Reeve (2007[14]), when used properly, IRT can be a powerful tool for the development of a questionnaire, and its evaluation and refinement, resulting in precise, valid and relatively succinct instruments. For Reise et al. (2005[40]), IRT is a group of mathematical models used to classify items on a scale, and to evaluate the quality of an item, serving to produce psychometric properties about the construct. Given these assumptions, the purpose of this study was to develop and validate an instrument for the evaluation of tablet arm chairs using IRT based on a construct of ergonomic requirements and focused on user perceptions.

### Ergonomic requirements

A key issue to be resolved in the initial developmental stage is the scope or generality of the target construct, thereby deeming it necessary to review the relevant literature to see how others have approached the same problem (Clark and Watson, 1995[11]). In this study, the latent trait of the instrument is the suitability of ergonomic requirements (physical) of a tablet arm chair according to the perception of the user.

In accordance with the International Ergonomics Association (2000[19]), ergonomics is a scientific discipline related to the understanding of the interactions between human beings and other elements of a system, and to the application of theories, principles, data and methods to projects for improving human well-being and the global performance of a system. Moreover, ergonomics is divided into three domains: physical, cognitive and organizational. In this study, the items were prepared with a focus on the physical issues, which for the International Ergonomics Association (2000[19]) are related to anatomical, anthropometric, physiological and biomechanical characteristics related to physical activity. The study also includes relevant topics such as the posture adopted, the handling of materials, repetitive movements and musculoskeletal disorders caused by work. 

Since there is no specific norm for tablet arm chairs, the items relating to ergonomic requirements were based on the following physical specifications described in ABNT Technical Norm 14006 - School furniture - Chairs and tables for educational institutions (Associação Brasileira de Normas Técnicas, 2008[4]): limits of height, width and seat angle; size of the backrest and desk surface; depth of seat and desk; user accessible parts should not have sharp projections, recesses or perforations; the surfaces of the desk, seat and backrest should not have a roughness above the permitted limit or sharp edges; the feet for the desk and chair should rest perfectly on a flat surface; the desk should not have more than 30 shine units; and hardness. The same norm determines that the desk should be high enough that there is free space for a user's leg movements; desk tops manufactured with polymers can only have minor molding deformations on their surface; the shape of the front edge of the seat should be rounded. 

Various tests for tables are recommended by ABNT NBR 14006:2008 (Associação Brasileira de Normas Técnicas, 2008[4]) including those for: vertical and horizontal static load, support of load, vertical impact, horizontal fatigue, toppling and stability. They indicate that school furniture should have a physical structure that is adequate for supporting body weight while providing stability in response to movements made by users. 

Other ergonomic requirements were found within the literature: the seat should not be so high that it compresses the soft tissue of the underside of the thighs (Kroemer and Grandjean, 2001[25]), thus creating pressure that interferes with venous return of blood from the lower limbs and possibly causing discomfort/problems in thighs, knees and feet (Mandal, 1993[27]); the seat should not have excess curves and moldings, because these may impede movement of the user and ventilation of the body (Ministério da Educação e Cultura, Brazil, 1982[31]); a seat with overly stiff upholstery causes increased concentration of pressure on the region of the gluteal tuberosity, occasioning fatigue and pain (Iida, 2005[18]); the material that is in contact with the student's body should be a poor heat conductor (Bergmiller, 1999[6]); a seat used for working should be lined with anti-slip material to prevent the user from slipping to the front (Iida, 2005[18]); a large bucket seat allows students to move and shift their posture as needed (Wulsin Jr., 2013[50]).

According to Mandal (1981[28]), the change of posture from standing to sitting causes a change in the spine: the 90 degrees hip joint moves to 60 degrees, while the remaining 30 degrees come from the flattening of the lumbar curve. Makhsous et al. (2003[26]) reported that sitting may induce posterior rotation of the pelvis, reduction of lumbar lordosis, and increases in muscle tension, disc pressure, and pressure on the ischium and coccyx, which may be associated with low back pain. Thus, the backrest should be vertically convex, to support normal lumbar lordosis, and transversally concave to support the anatomy of the spinal column and offer lateral support to the trunk (Chaffin et al., 2006[9]); and the backrest should be positioned below the scapulas, or at most on the upper edge of the scapulas (Gouvali and Boudolos, 2006[17]), to facilitate mobility of the trunk and arms (Oborne, 1995[33]), and should have a free space of 150-200 mm between the part below the lumbar support and the seat (Brandimiller, 2002[7]) to accommodate the gluteal region (Ministério da Educação e Cultura, Brazil, 1982[31]).

Chairs that are very tall and deep, high desks, and a back-to-desk distance inadequate for the anthropometry of the user, have negative effects on the seated posture, especially for reading and writing activities (Panagiotopoulou et al., 2004[34]). One of the specific educational criteria is that the environment should promote mobility of the furniture, which is essential for teaching and learning (Bergmiller, 1999[6]). In addition, school furniture should not make noise when it is being used, because this can be disturbing (Panagiotopoulou et al., 2004[34]; Wulsin Jr., 2013[50]).

In relation to the material holder, it is recommended that it does not interfere with posture when storing school materials (Khanam et al., 2006[23], 2006[22]); when it is under the seat, it should be able to hold material and have some form of protection at the rear to prevent the material from falling (Ministério da Educação e Cultura, Brazil, 1982[31]). According to Bergmiller (1999[6]), there should be enough space to hold materials so that a student can work in an organized manner.

## Materials and Methods

### Participants

The study involved the voluntary participation of 1,633 individuals (students and professors) in different phases of the study, which were selected in an intentional manner. Each participant signed a consent form and the study was approved by the local Ethics Committee, Federal University at Santa Catarina, Brazil.

### The study steps 

The items of the instrument were developed by the authors in a series of steps:

1. Development of the pool of items based on the construct of the instrument - ergonomic requirements in the physical domain of ergonomics.
2. A pilot study (n = 26 university students) - aimed to test the instrument with a smaller group of students by checking the understanding of the items.
3. Restructuring of the instrument as the pilot study considerations.
4. Semantic validation (n = 430 university students) - aimed to test the instrument with a larger group of students by checking the understanding of the items.
5. Restructuring instrument as considerations made on the validity of semantics.
6. Content validation (n = 11 professors) - specialists have found that the items were pertinent to the construct.
7. Restructuring instrument as considerations made in the content validation.
8. Construct validation (n = 1,166 university students) - application of IRT. Where each item received a score, where appropriate, the item was eliminated when it did not meet the pre-established criteria.

### Development of the items

The psychometry techniques recommended by Pasquali (1998[36]) were used to construct the items that are part of the instrument. The items were based on scientific literature in the field of ergonomics (Iida, 2005[18]; Chaffin et al., 2006[9]), technical norms for office (Associação Brasileira de Normas Técnicas, 2006[3]) and school furniture (chair and table) (Associação Brasileira de Normas Técnicas, 2008[4]), papers about school furniture (Milanese and Grimmer, 2004[30]), seated posture (Corlett, 2008[12]), tablet arm chairs (Dianat et al., 2013[13]; Khanam et al., 2006[23], 2006[22]; Thariq et al., 2010[46]), and by the evaluations of university students and specialists (PhD professors) who took part in the study.

The items were placed in the instrument according to the parts that compose the tablet arm chair, and considered the subsystems - seat, backrest, tablet arm, tablet arm extension and material holder (Figure 1(Fig. 1)) - and general items were included about school furniture. To help the respondents understand the items, drawings that clarify the ergonomic requirements to which each item refers were placed in the instrument used to validate the construct. 

Since the instrument is designed to measure users' perceptions about the suitability of the ergonomic requirements, in the steps of the pilot study and construct validation, respondents were asked to pay attention to his or her own body, the tablet arm chair in which he or she was seated, do what was asked (the task) and respond to the item by choosing from the optional responses in order to indicate their understanding and agreement with the items. In the content validation, specialists selected from optional responses to indicate pertinence.

For Pasquali (1998[36]), the development and validation process of the instrument occurs in four steps: pilot study, semantic validation, content validation and, finally, construct validation. These are described below.

### Pilot study

In the pilot study, 26 university students with a mean age of 23.3 years (range 19 to 37 years) and from five different courses analyzed the items of the instrument. The data collection was previously scheduled with the students and conducted in classrooms with three models of tablet arm chairs. Each item of the instrument was analyzed jointly by the group of students in a discussion format, and their semantics were verified to determine that they were correctly understood, and if there was a need to change, include or exclude a word and/or item. 

### Semantic validation

To validate the semantics, the instrument resulting from the pilot study was evaluated to 430 university students with a mean age of 22.4 years (range 18 to 61 years), from five institutions of higher education in Florianópolis, Santa Catarina (public and private) and from different courses and class periods (morning, afternoon and night). The collections were scheduled with professors who taught in classrooms with tablet arm chairs (totaling 13 different models) and who agreed to provide the last 30 minutes of their class to apply the instrument. Each student received an instrument, individually analyzed each item to confirm their understanding and to indicate if he or she understood completely, partially or not at all, and also made written suggestions for changes. 

### Content validation

After the reformulation of the instrument, the content validation step began, in which the instrument was sent to 44 professors who are specialists in the issue, and were selected by using Brazil's online Lattes platform, which provides the curriculums of university professors. Of these, 11 professors agreed to evaluate the instrument in terms of the pertinence of the items of the construct. To analyze the results, criteria developed by Pasquali (1998[36]) were used, which recommended that an item remain in the instrument if it had agreement from 80 % of the specialists. Andrade (2007[1]) mentions that items found to be pertinent should remain in the instrument; items considered indifferent can remain or be excluded at the discretion of the researcher, while the items found to be impertinent should be excluded. 

### Construct validation

Item Response Theory (IRT), also known as latent trait theory, is a measurement model that is an alternative to the true score theory test. IRT makes stronger assumptions than the classical test theory and, in many cases, provides results that are proportionately stronger (Kline, 2005[24]). So this study used IRT to validate the items developed in this instrument. IRT describes a set of mathematical models to measure latent traits (that is, individual profile characteristics that cannot be measured directly). The models use a set of items to construct a scale such that the latent trait of the respondent and item difficulty can be compared (Embretson et al., 2000[15]).

The use of IRT allows us to evaluate the latent traits of each item and of the instrument by means of parameters generated for each item (Reise et al., 2005[40]). To validate this instrument, the parameters used were: discrimination (*a*) and difficulty (*b*). According to Reise et al. (2005[40]), parameter *a *is proportional to the derivative of the tangent of the curve at the point of inflection and has the function of distinguishing individuals, because the higher the value of this parameter, the more the item differentiates respondents with different latent trait levels. Meanwhile, parameter *b *represents the position of the item on the scale, showing if it is difficult or easy for this item to be present among the respondents to the instrument. 

To calibrate the items, Edelen and Reeve (2007[14]) recommend that there be respondents to the items in each response category so that the parameters of the items can be estimated on the various levels of the scale. The same authors suggest that the sample be ≥ 200 respondents in order to decrease the estimated standard error.

**Figure 2:**
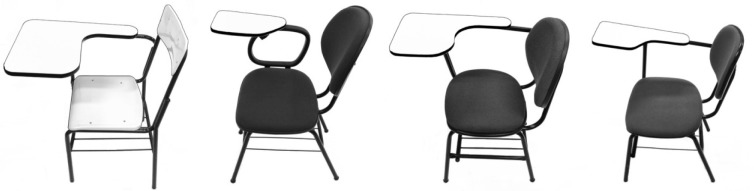
Types of tablet arm chairs used in the validation of the construct

To encompass and represent the population (of students and tablet arm chairs) being studied, four types of tablet arm chairs (Figure 2(Fig. 2)) were selected to be part of the construct validation step. The tablet arm chairs had different seat and backrest materials and the sizes of the tablet arm and of the tablet arm extension also varied. There were more than 200 respondents for each type of tablet arm chair.

The criteria adopted for selecting the sample included collecting data from students in different courses, years and time periods, with the understanding that in this way the opinions about the tablet arm chairs would be more diverse. 

The construct validation occurred with 1,166 university students with a mean age of 22.5 years (range 18 to 58 years) from two institutions of higher education. The scheduling and data collection were similar to that used for the semantic validation, although the collections were conducted in classrooms that had at least one model of the four tablet arm chairs selected for the study (Figure 2(Fig. 2)).

In this phase, each student evaluated the tablet arm chair in which he or she was seated using the instrument developed in this study. Each item was evaluated separately and, using a Likert scale, received one of the four categories of responses proposed by Araujo et al. (2009[2]): disagree completely, disagree, agree and agree completely.

To conduct the statistical analysis, the software MULTILOG^® ^*for Windows*^® ^2003 (Skokie, IL, USA) was used and Samejima's (1969[41]) graded response model was applied. The estimates of the discrimination (*a*) and difficulty (*b*) parameters were obtained in the scale (0, 1). 

This model assumes that an order can be established among the categories of responses to an item. In this way, the probability that an individual *j *with ability θ will choose a category *k *(0, 1, 2, 3) is given by:


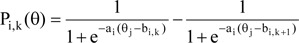


in which the item that has four categories has three values of the difficulty parameter (*b_1_, b_2, _b_3_*), in addition to the discrimination parameter (*a*)*. *The parameters of the items and the latent trait of the respondent will determine the probability that he or she would choose each one of the categories for a given item. It should be noted that this model should have an order between the parameters of difficulty for a given item, which is: b_i,1_ ≤ b_i,2_ ≤ b_i,3_.

Some authors use as a criterion for exclusion of an item a value for parameter *a *for dichotomic responses < 0.70 (Tezza et al., 2011[45]; Trierweiller et al., 2012[47]). Nevertheless, for studies with graded responses (polytomic), no reference values were found in the literature. In this study, the parameter *a *< 0.65 was adopted as a criterion for exclusion of an item of the instrument. This value was defined due to the decision to maintain two items from the subsystem “material holder” that had parameters for *a* between 0.65 and 0.70, considering that this subsystem has few items. Therefore, we considered that the items with low discrimination parameters (a < 0.65) provided little information about the suitability of the table arm chair ergonomic requirements and was not worth being in the instrument.

## Results

In all the steps of the analysis of the items, both in the theoretical and empirical steps of the validation process, the instrument was composed of 57 items, modifying only the quantity of items in each subsystem, given that, in the final version of the instrument (Appendix A) after the statistical analysis, 12 items were excluded (Table 1(Tab. 1)).

After the students evaluation in the pilot study, six items referring to the subsystem seat (depth [two items]; side edges, inclination and texture [three items] and texture [one item]), one about the backrest (texture) and one about the tablet arm extension (texture) were excluded. Although, two items about the backrest (height and side edges) and six in the general topics of the tablet arm chair (stability, transportation and accessories [two per topic]) were added to the instrument. The exclusions were due to subjectivity and dependence between the items, while the inclusions were due to the suggestions of respondents. 

The result of the semantic validation by the 430 students led to adjustments in the wording used in 33 of the 57 items, so that respondents would better understand them, although no item was excluded. 

The content validation found that all of the items should remain in the instrument, because more than 80 % of the specialists considered the items pertinent to the construct “suitability of the ergonomic requirements of a tablet arm chair,” although 26 items underwent some change in their structure after this step. 

The validation of the construct was conducted by the calibration process for the 57 items of the instrument, which consisted of estimating the parameters *a*, given that 12 items had a value for parameter *a *that was considered low (< 0.65). These items were removed from the calibration of the instrument one after the other and in growing order of the values of parameter *a*. The results of the discrimination parameters *a *and of the three parameters of difficulty (*b_1_, b_2 _*and *b_3_*) of the items with the respective items after the final calibration are presented in Table 2(Tab. 2).

The results presented average discrimination parameters of 1.226 and average difficulty parameters of -2.2, -0.72 and 2.03. The Cronbach's alpha reliability coefficient of the final instrument was 0.95, a very high value, which validates the instrument.

**Figure 3:**
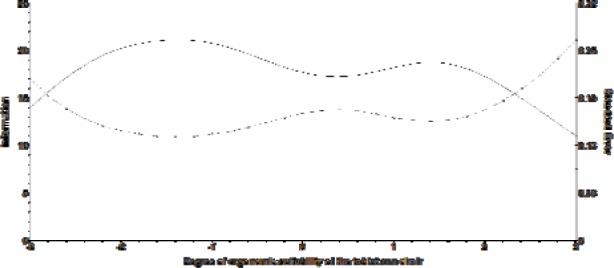
Total information curve for the instrument in the scale (0,1) (information from the test: continuous line; standard error: dotted line)

The set of items provides a function of information of test, and from this function is determined the standard error of the measurement, which results in a graph that represents the instruments' curve of total information (Figure 3(Fig. 3)). 

The information curve presents the amount of information in each point of the scale. The standard error curve shows that the instrument will generate more accurate estimates (low standard errors < 0.19) of the degree of ergonomic suitability of the tablet arm chair in regions with high information. One can see in Figure 3(Fig. 3) that the instrument is able to generate good estimates in the range of 2 standard deviations above and below the mean (zero), which is a fairly wide range in the scale (0, 1). Accordingly, the developed instrument is suitable for evaluating tablet arm chairs and users with different characteristics, that is, tablet arm chairs with low, average or high ergonomic suitability.

To verify the latent trait interval that the instrument developed is capable of measuring, all the items were completed with positive answers in terms of suitability of the tablet arm chair, and with all the items with negative responses, the result of the statistical analysis indicated that the instrument was able to generate estimates for the latent trait at an interval of -3.56 to 3.56.

## Discussion

The items presented in the results of this article are based on a statistical analysis, and it was determined that items that did not have acceptable parameters should be excluded. To do so, the following factors were considered: if the item provided little information about the requirement to be measured, if the item was poorly formulated (ambiguous or confusing) and also if it was necessary to maintain the item in the instrument to measure the latent trait of the subsystem of the tablet arm chair. 

In the instrument's order of presentation (Table 2(Tab. 2)), the first item to be eliminated was item 02 because it had a parameter *a *< 0.65 and also because it referred to the same requirement contemplated in item 01 - seat height, which would not harm the scope of the instrument.

Item 08 refers to the shape of the front edge of the seat, which should be rounded (Associação Brasileira de Normas Técnicas, 2008[4]) and soft (Chaffin et al., 2006[9]) in order to meet the ergonomic requirements. The responses to this item may have suffered interference from the height requirement for the seat, because, according to Iida (2005[18]), when a seat is high for the user and the soles of the feet are not completely supported on the ground or on a support, the seat can apply pressure to the rear thigh muscles.

The items referring to the heat caused by the seat material (12) and the backrest (23) perceived by the user may be related with many variables, such as the sex of the user. A study by Yamtraipat et al. (2005[51]) reported that a group of men perceived a lower average neutral temperature than a group of women, and highlighted that women report using more clothes than men. Another point that may interfere in the response to these items is the weather conditions, season of the year and region of the country where the instrument is applied, in addition to ambient conditions in the room itself (whether it has fans or air conditioning). According to Yamtraipat et al. (2005[51]), various factors can affect the sensation of thermal comfort, both quantitative and qualitative. This study found that being accustomed to the use of air conditioning at home and a high level of schooling made users in the air-conditioned rooms prefer lower temperatures, although, on average, the difference between one temperature found comfortable and the other, is very small (< 1 °C). A study by Song et al. (2007[44]), upon measuring the superficial scrotal temperature in 10 men after being seated for 120 minutes, found that there was no correlation of this variable with the thickness of the chair cushions; nevertheless, this factor was influenced by the ambient temperature in the artificially air-conditioned room (18 and 26 °C).

Item 16 was eliminated because most of the students (90.3 %) found that the backrest did not touch the buttocks during use, thus this item had little information, not discriminating the students and the chairs. There is a lack of studies that investigate the perception of user discomfort from chairs with full backrests and with curvature to accommodate the buttocks and even of tablet arm chairs in which the backrests only support the thoracic spine. Some studies have contradictory findings, reporting that different regions of the body are supported on the backrest. According to Khanam et al. (2006[22]), the backrest should support the weight of the body from the upper region of the lumbar up to the height of the acromion, while other studies do not always provide details about the regions supported (Vergara and Page, 2000[49]) and only mention that the backrest serves to support the lumbar and dorsal region.

Item 19 refers to the shape of the edges of the backrest. According to the Associação Brasileira de Normas Técnicas (2008[4]), the rays of curvature of the corners of the edges both of the desktop and of the seat and backrest should be 2.5 mm (rounded). This item did not have an acceptable parameter of discrimination because the majority of the respondents (71.3 %) agreed or completely agreed that the shape of the edges of the backrest did not place pressure on the back, being an ergonomic requirement found in most of the tablet arm chairs, and providing little information, and for this reason it did not remain in the instrument.

Items 33 and 34, which referred to the tablet arm, were excluded, because 61.3 % and 90 % of the respondents agreed or completely agreed, respectively, that the tablet arm reflected light and was smooth. It was observed during the data collection that some students complained that the sheets of paper slipped on the tablet arm because it was very smooth. One of the ergonomic requirements set by the Associação Brasileira de Normas Técnicas (2008[4]) is that the tablet arm not have recesses, although it was found that the way that the item was prepared could allow a dual interpretation, with the tablet arm suitable because it is smooth and, at the same time, unsuitable because it does not have enough adherence for a sheet of paper to remain on it. Item 33 may not be precise due to the fact that users did not verify this requirement in the tablet arm chair because most of the tablet arms are small and materials cover nearly the entire tablet arm. 

Although requirements concerning safety and finish in the regulatory norm (Associação Brasileira de Normas Técnicas, 2008[4]) determine that parts that are accessible to users of school furniture should not have sharp protrusions, recesses or perforations, item 44 of this instrument was excluded. The low values of the parameters found in this item can be justified, because according to Khanam et al. (2006[23]), the students do not use the material holder because it is situated in an inadequate location (at the height of the feet) and users have to adopt an uncomfortable posture when storing material. Khanam et al. (2006[22]) suggest that the material holder can be fixed next to the seat, so the student can handle books in the sitting position and without using awkward postures.

Finally, in relation to the general items of the tablet arm chair, it can be concluded that items 55 and 56 could have been grouped into one, because they became very specific by addressing the ease of transportation in terms of shape and weight of the tablet arm chair, and may have caused doubt among respondents, which led to low values of the parameters. Meanwhile, item 57 showed that the majority of the students (93.4 %) found that the tablet arm chair does not have a place to store small items, a requirement that is absent in the types of tablet arm chairs studied and for which reason this item was excluded.

Regarding the items with higher parameters *a*, which remained on the instrument, we highlight the items that relate to the general aspects of the tablet arm chair, more specifically the stability (items 50 and 51). Although the Brazilian technical standard on school furniture manufacturers recommend to conduct several tests, two of them, toppling and stability (Associação Brasileira de Normas Técnicas, 2008[4]), it was noticed by the students surveyed that not all the requirement were always met by the manufactures.

The most discriminant items on subsystem seat (6 and 7) concern about the shape of the seat surface and the distribution of the body weight in the seat. According to Khanam et al. (2006[22]), seating furniture for classroom shall support the body weight and enable postural movement and circulations. It is not perceived by most participants in the study, so the parameters *a* were high, indicating that the items have lots of information and must remain in the instrument.

Items 21 and 26 achieved the highest parameters *a* in the subsystem "backrest". They refer to the inclination of the backrest and the angle between the seat and the backrest. For Pheasant (1986[37]), the purpose of seating furniture is to provide stable body support in a posture that is comfortable over a period of time, physiologically satisfactory and appropriate to the task or activity.

Items 28 and 36 were the most discriminant ones in subsystem "tablet arm". They measure the aspects related to stability and if there is enough space between the tablet arm and one's thighs to allow free movement of the legs. According to Khanam et al. (2006[23]), the arm tablet fixed on to the right side of the chair is designed to facilitate writing. Regarding the space, Khanam et al. (2006[23]) found that the students surveyed preferred high furniture that could be adjusted and with enough space for the thighs and legs. However, it was found that several students realized that the tablet arm as unstable and that there was not enough space to accommodate their legs below it.

Finally, items 39 and 40 of the subsystem "tablet arm extension", that include the tablet arm extension width and if the extension supports the forearm without slipping, respectively, obtained the best parameters *a*. A factor that may have caused the best parameters *a *for most items is the discrepancy between the students' anthropometric characteristics and the dimensions of school furniture, emphasized by several studies (Chung and Wong, 2007[10]; Dianat et al., 2013[13]; Thariq et al., 2010[46]). Likewise, the comfort and the functional utility of school furniture depend on its design (physical) in relation to the physical structure of the human body and biomechanics (Khanam et al., 2006[23]).

Babbar et al. (2002[5]) mention that it is not sufficient for companies to supply products with technical excellence, and suggest that products must be easy to use and should meet users' needs in relation to the activities that they want to conduct and the context into which the product is inserted. For this reason, studies are required to identify the needs and reality of the students who use tablet arm chairs, thus promoting the development of ergonomically suitable school furniture.

## Conclusion

It can be concluded that the validation process for the instrument was successful in all the steps (theoretical and practical) and that after the statistical analysis, the group of remaining items presented very good consistency, containing enough information to evaluate the suitability of the ergonomic requirements for tablet arm chairs.

The instrument resulting from this study can be used to evaluate this type of furniture during the purchasing process. The analysis can be made available via the Internet, where public and private universities could use a site to benefit from the instrument when making purchasing decisions. Manufacturers of tablet arm chairs can also use the instrument to apply ergonomics for correctional purposes, because the instrument allows for identification of the elements that do not meet the ergonomic requirements necessary for attending user needs or the subsystem that requires improvement. The instrument could also be part of a step of a regulatory norm concerning the comfort and suitability of this type of furniture. 

It is suggested that the following procedures be used when applying the instrument: have at least six evaluators of average anthropometric stature (three men and three women); conduct tasks in which the evaluators remain seated in the tablet arm chair, listening, reading and writing for at least 30 minutes before beginning the ergonomic evaluation of the tablet arm chair. 

The instrument was originally developed in Portuguese. It is thus recommended that before applying the instrument, the researcher validate it in the local language, particularly in relation to the terms used for the items (semantic), or else the instrument may be incomprehensible because of cultural and language differences. 

Future studies can be conducted to develop and include new items in the instrument, and to explore other ergonomic requirements for tablet arm chairs and for using IRT as an objective and consistent way to evaluate the suitability of ergonomic requirements according to the anthropometric characteristics of users. 

## Acknowledgements

We would like to thank CNPq and CAPES for the doctoral grants.

## Conflict of interest

The authors declare that they have no conflict of interest.

## Supplementary Material

Appendix A

## Figures and Tables

**Table 1:**
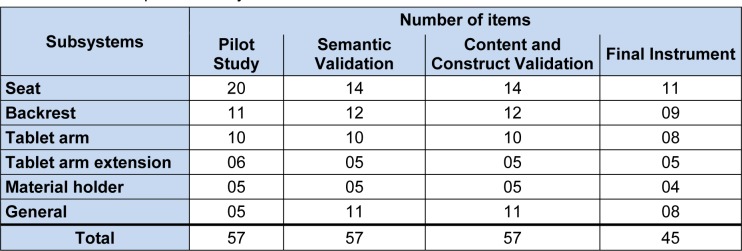
Quantity of items in the different steps of the development and validation process of the instrument in the respective subsystems

**Table 2:**
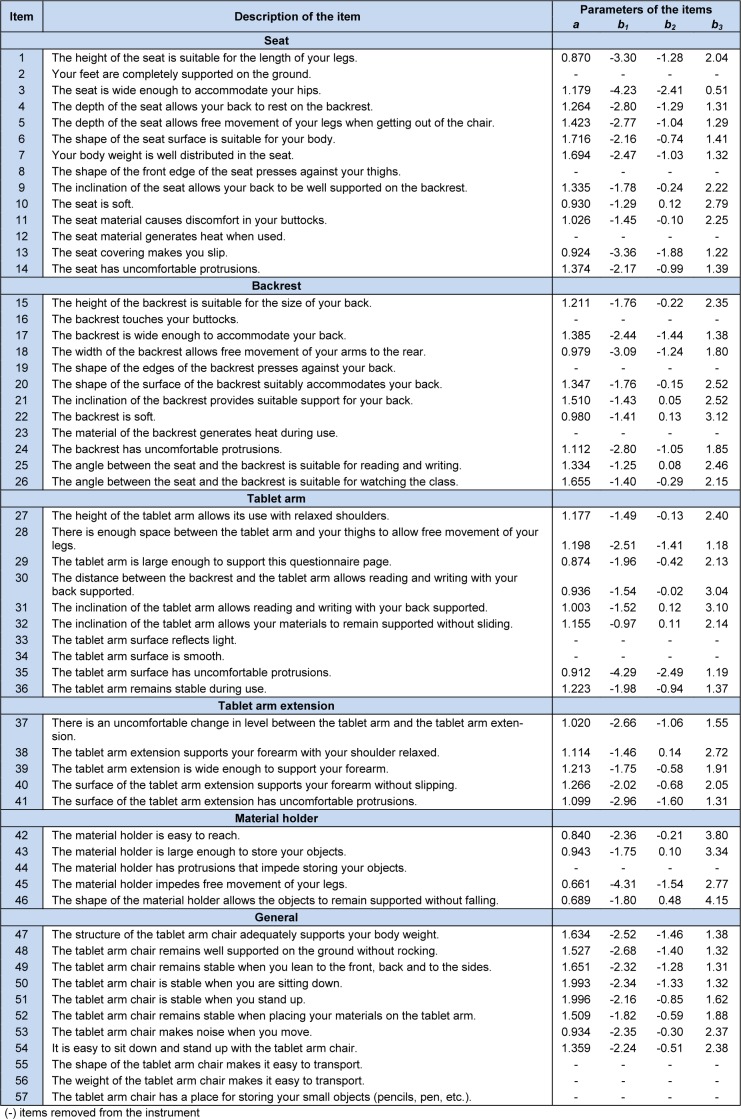
Description of the items and estimates of the parameters of discrimination and difficulty
