# Supplementary material for: Development and validation of instrument for ergonomic evaluation of tablet arm chairs
Source: EXCLI J. 2016 Nov 7;15:671–86. doi: 10.17179/excli2016-568 (PMC5318684; doi:10.17179/excli2016-568)
Supplement: Appendix A [file EXCLI-15-671-s-001.pdf]

EXCLI Journal 2016;15:671-686 – ISSN 1611-2156
Received: August 07, 2016, accepted: October 12, 2016, published: November 07, 2016

Appendix A – Instrument for evaluation of the ergonomic requirements of a tablet arm chair

ERGONOMIC EVALUATION OF A TABLET ARM CHAIR

Respondent data:

Age:________ Sex: ( ) M ( ) F Weight (kg):___________ Height (m):_________

Course:____ Year:______________

Type of tablet arm chair:____

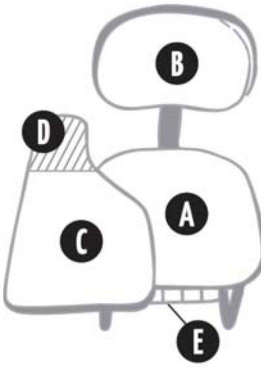

Instructions:

1 – The analysis of the tablet arm chair will take place in steps for each of the following parts:

(A) seat **(B)** backrest **(C)** tablet arm **(D)** tablet arm extension **(E)** material holder

2 – When responding to the questions you should be seated correctly on the hip bones with the spine erect.

3 – You should pay attention to your body and the chair in which you are seated.

4 – Read each question carefully and mark the response that represents your opinion with an x:

DC = disagree completely, **D** = disagree, **A** = agree, **AC** = agree completely, and **NA** = not applicable (if this part of the chair does not exist).

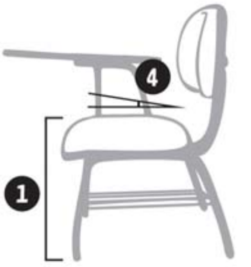

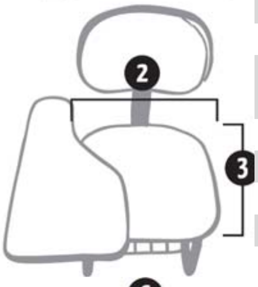

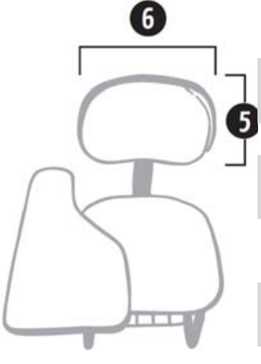

Answer the questions according to your perceptions.

(A) Seat

	DC	D	A	AC	NA
1. The height of the seat **(1)** is suitable for the length of your legs.					
2. The seat is wide **(2)** enough to accommodate your hips.					
3. The depth of the seat **(3)** allows your back to rest on the backrest.					
4. The depth of the seat allows free movement of your legs when getting out of the chair.					
5. The shape of the seat surface is suitable for your body.					
6. Your body weight is well distributed in the seat.					
7. The inclination of the seat **(4)** allows your back to be well supported on the backrest.					
8. The seat is soft.					
9. The seat material causes discomfort in your buttocks.					
10. The seat covering makes you slip.					
11. The seat has uncomfortable protrusions.					

(B) Backrest

	DC	D	A	AC	NA
12. The height of the backrest **(5)** is suitable for the size of your back.					
13. The backrest **(6)** is wide enough to accommodate your back.					
14. The width of the backrest allows free movement of your arms to the rear.					
15. The shape of the surface of the backrest **(7)** suitably accommodates your back.					
16. The inclination of the backrest **(8)** provides suitable support for your back.					
17. The backrest is soft.					
18. The backrest has uncomfortable protrusions.					

19. The angle between the seat and the backrest **(9)** is suitable for reading and writing.					
20. The angle between the seat and the backrest is suitable for watching the class.					

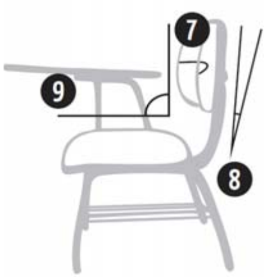

**(C) Tablet arm**	DC	D	A	AC	NA
21. The height of the tablet arm **(10)** allows its use with relaxed shoulders.					
22. There is enough space between the tablet arm and your thighs **(11)** to allow free movement of your legs.					
23. The tablet arm **(12)** is large enough to support this questionnaire page.					
24. The distance between the backrest and the tablet arm **(13)** allows reading and writing with your back supported.					
25. The inclination of the tablet arm **(14)** allows reading and writing with your back supported.					
26. The inclination of the tablet arm allows your materials to remain supported without sliding.					
27. The tablet arm surface has uncomfortable protrusions.					
28. The tablet arm remains stable during use.					

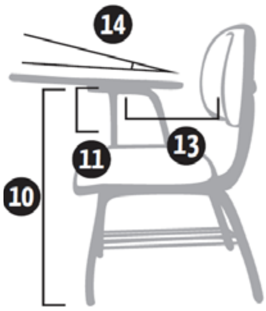

**(D) Tablet arm extension**	DC	D	A	AC	NA
29. There is an uncomfortable change in level between the tablet arm and the tablet arm extension **(15)**.					
30. The tablet arm extension supports your forearm with your shoulder relaxed.					
31. The tablet arm extension **(16)** is wide enough to support your forearm.					
32. The surface of the tablet arm extension supports your forearm without slipping.					
33. The surface of the tablet arm extension has uncomfortable protrusions.					

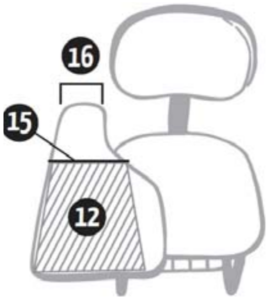

**(E) Material holder**	DC	D	A	AC	NA
34. The material holder is easy to reach.					
35. The material holder is large enough to store your objects.					
36. The material holder impedes free movement of your legs.					
37. The shape of the material holder allows the objects to remain supported without falling.					

**General**	DC	D	A	AC	NA
38. The structure of the tablet arm chair adequately supports your body weight.					
39. The tablet arm chair remains well supported on the ground without rocking.					
40. The tablet arm chair remains stable when you lean to the front, back and to the sides.					
41. The tablet arm chair is stable when you are sitting down.					
42. The tablet arm chair is stable when you stand up.					
43. The tablet arm chair remains stable when placing your materials on the tablet arm.					
44. The tablet arm chair makes noise when you move.					
45. It is easy to sit down and stand up with the tablet arm chair.					
